# Supplementary material for: Population genomics of Bacillus anthracis from an anthrax hyperendemic area reveals transmission processes across spatial scales and unexpected within-host diversity
Source: Microb Genom. 2022 Feb 21;8(2):000759. doi: 10.1099/mgen.0.000759 (PMC8942019; doi:10.1099/mgen.0.000759)
Supplement: Supplementary material 1 [file mgen-8-0759-s001.pdf]

Supplementary Material

Supplementary Figures

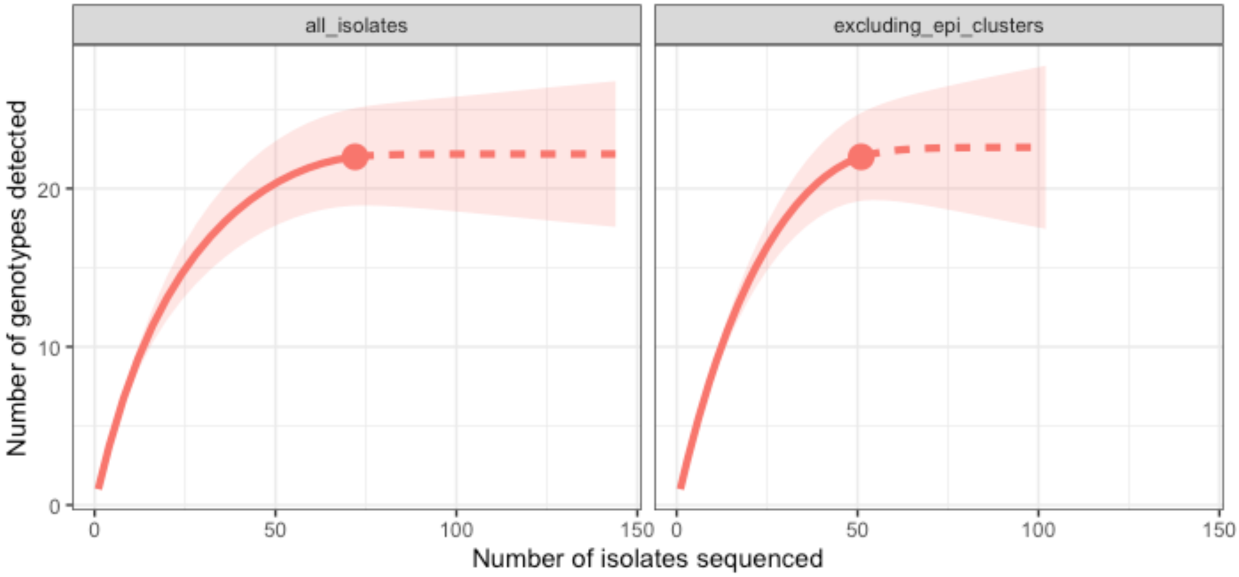

Fig S1. Rarefaction curve of *Bacillus anthracis* genotypic diversity within the study area. Based on inclusion of all isolates sequenced (n = 73, left) and on all isolates that were not sampled as part of an epidemiological cluster (n = 51, right), which might be expected to be non-independent. Results suggest genotypes within this population have been exhaustively sampled (i.e. that further sampling would not be expected to reveal additional genotypes). Figure generated in R package iNEXT [1].

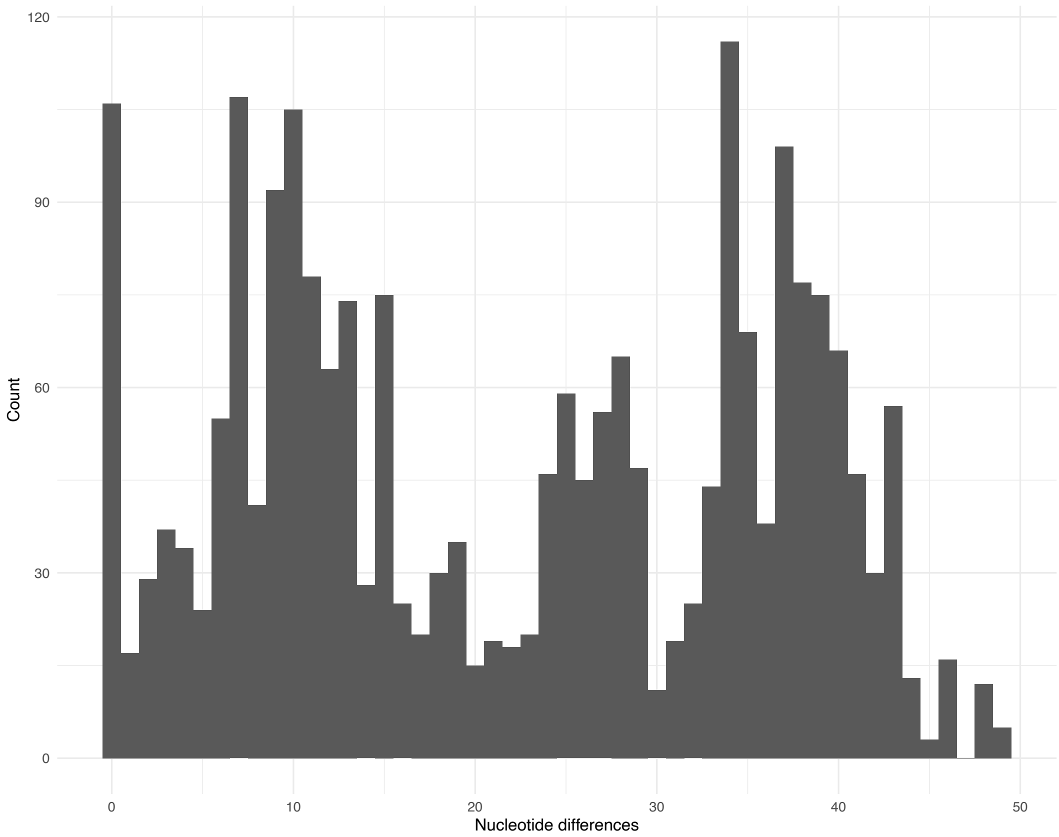

Fig S2. Histogram showing the relative frequency of pairwise nucleotide (SNP) differences among *B. anthracis* isolates. Along the x-axis are the numbers of pairwise nucleotide differences observed between each pair of 73 isolates from the Ngorongoro Conservation Area, northern Tanzania. The y-axis shows the number of times each of these pairwise differences were observed.

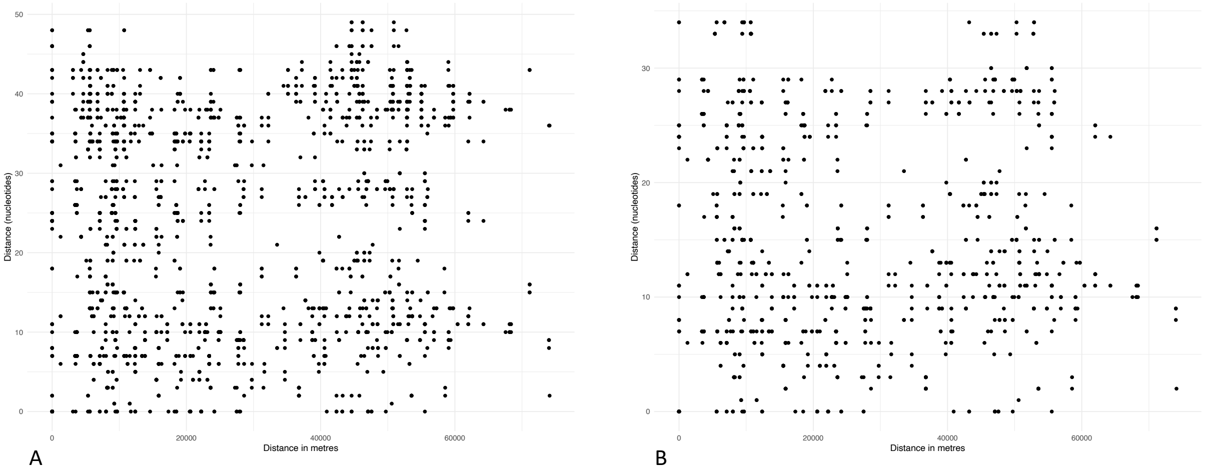

Fig S3. Scatter plots showing the number of nucleotide differences as a function of geographic difference between the sampling locations. Geographic distance (in meters) is shown on the x-axis versus nucleotide differences on the y-axis, with each point representing a pair of isolates. A) All *Bacillus anthracis* isolates from the study area. B) The same relationship is observed when limited to isolates from the dominant clade; this was done in order to account for deeper divergences potentially obscuring patterns.

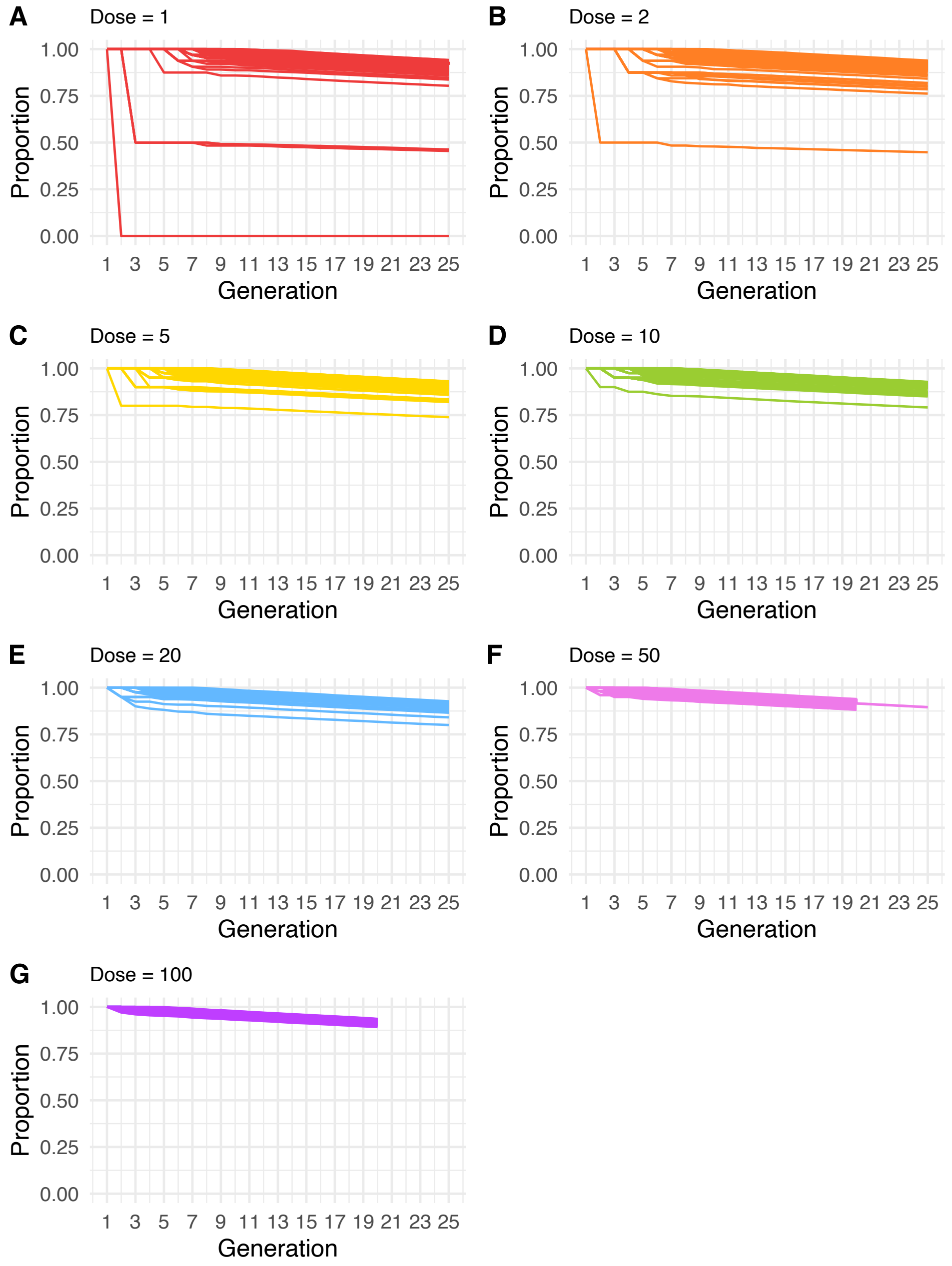

Fig S4. Proportion of simulated within-host populations identical to the inoculating genome over 25 generations. Populations were simulated from homogenous inoculating doses of varying size (A-G) and each line tracks a single simulated population through generations. Represented are 100 simulations run for 25 generations from doses 1, 2, 5 and 10; 50 simulations run for 25 generations (dose 20); 100 simulations run for 20 generations from doses 50 and 100 and 7 simulations run for 25 generations from inoculum dose of 50.

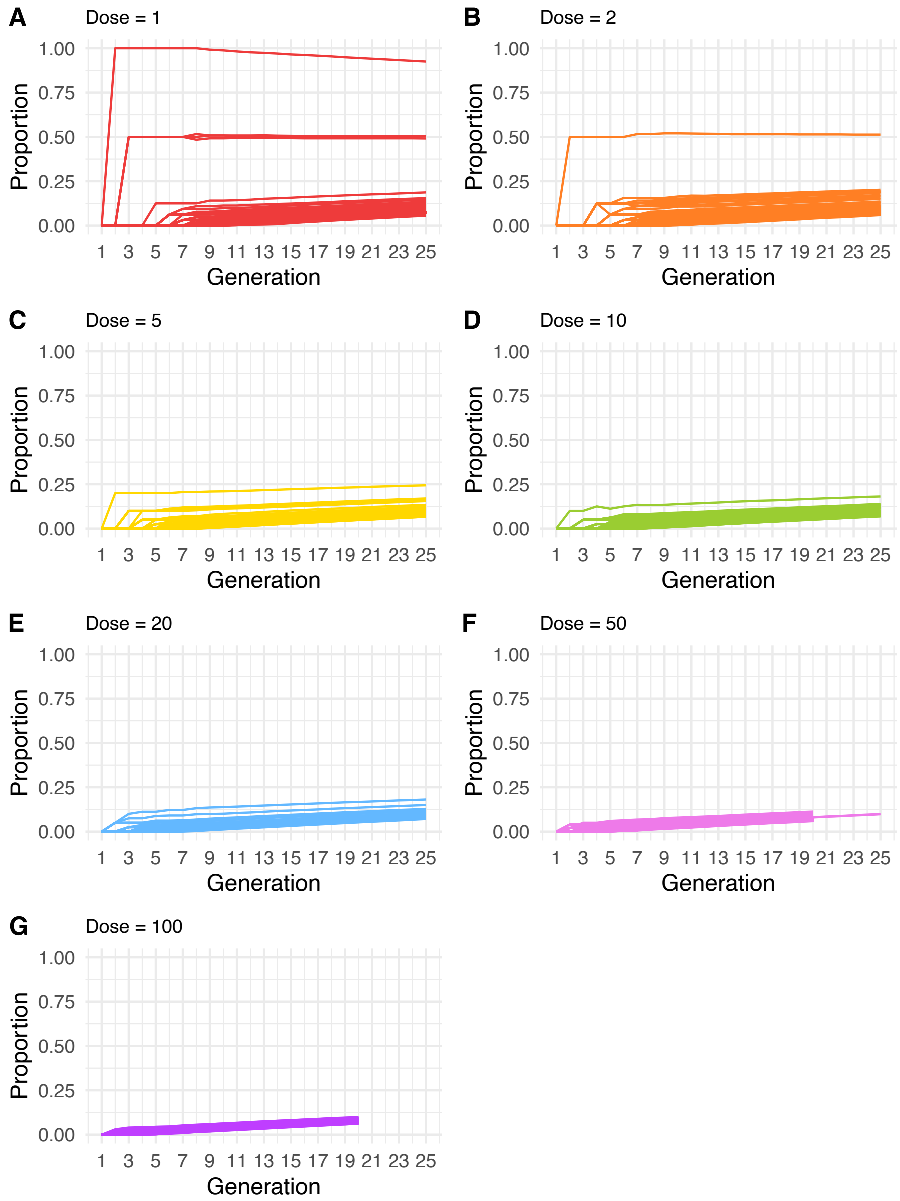

Fig S5. Proportion of simulated within-host populations differing from the inoculating genome by one nucleotide (SNP). Populations were simulated from homogenous inoculating doses of varying size (A-G) and each line tracks a single simulated population through generations. Represented are 100 simulations run for 25 generations from doses 1, 2, 5 and 10; 50 simulations run for 25 generations (dose 20); 100 simulations run for 20 generations from doses 50 and 100 and 7 simulations run for 25 generations from inoculum dose of 50.

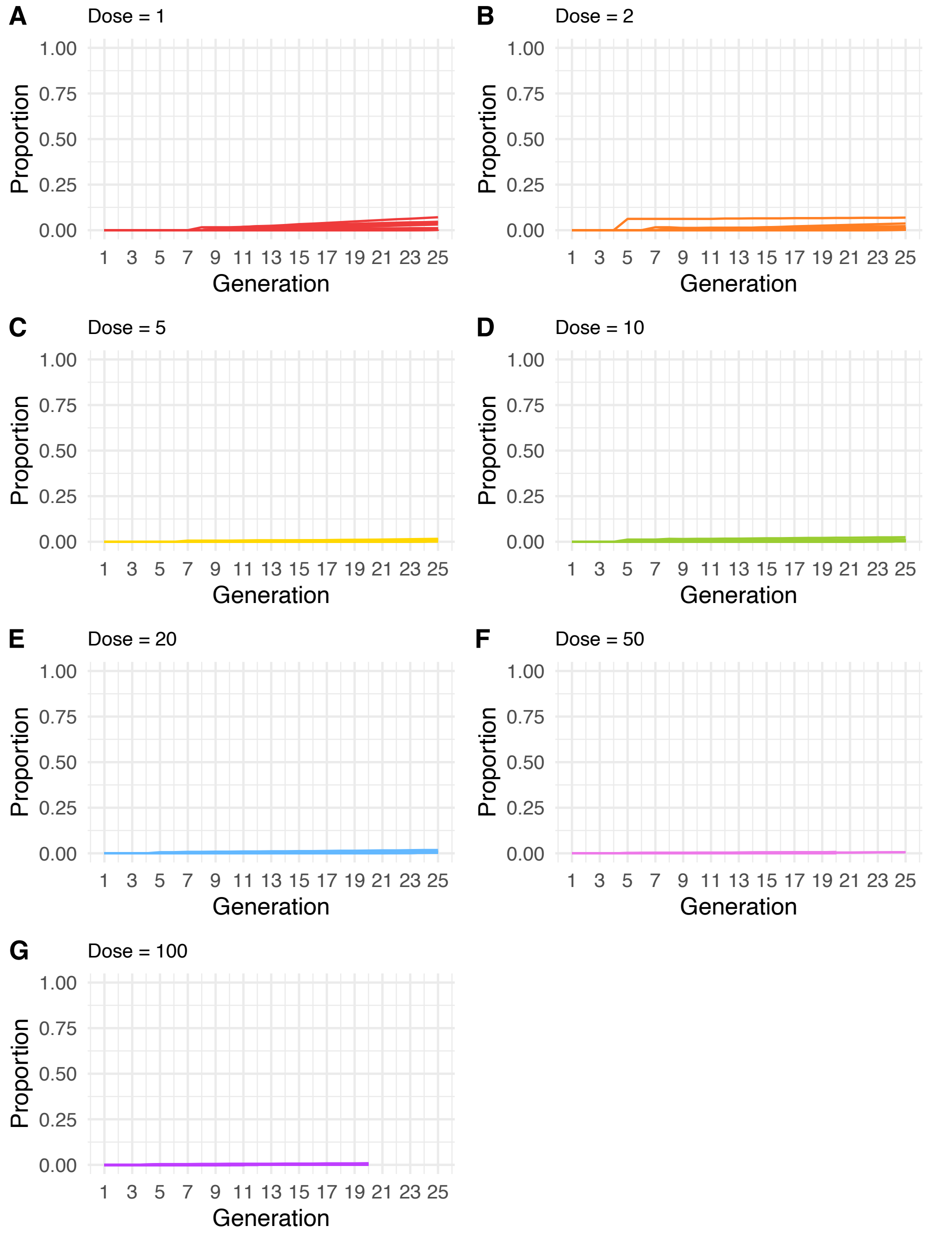

Fig S6. Proportion of simulated within-host populations differing from the inoculating genome by two nucleotides (SNPs). Populations were simulated from homogenous inoculating doses of varying size (A-G) and each line tracks a single simulated population through generations. Represented are 100 simulations run for 25 generations from doses 1, 2, 5 and 10; 50 simulations run for 25 generations (dose 20); 100 simulations run for 20 generations from doses 50 and 100 and 7 simulations run for 25 generations from inoculum dose of 50.

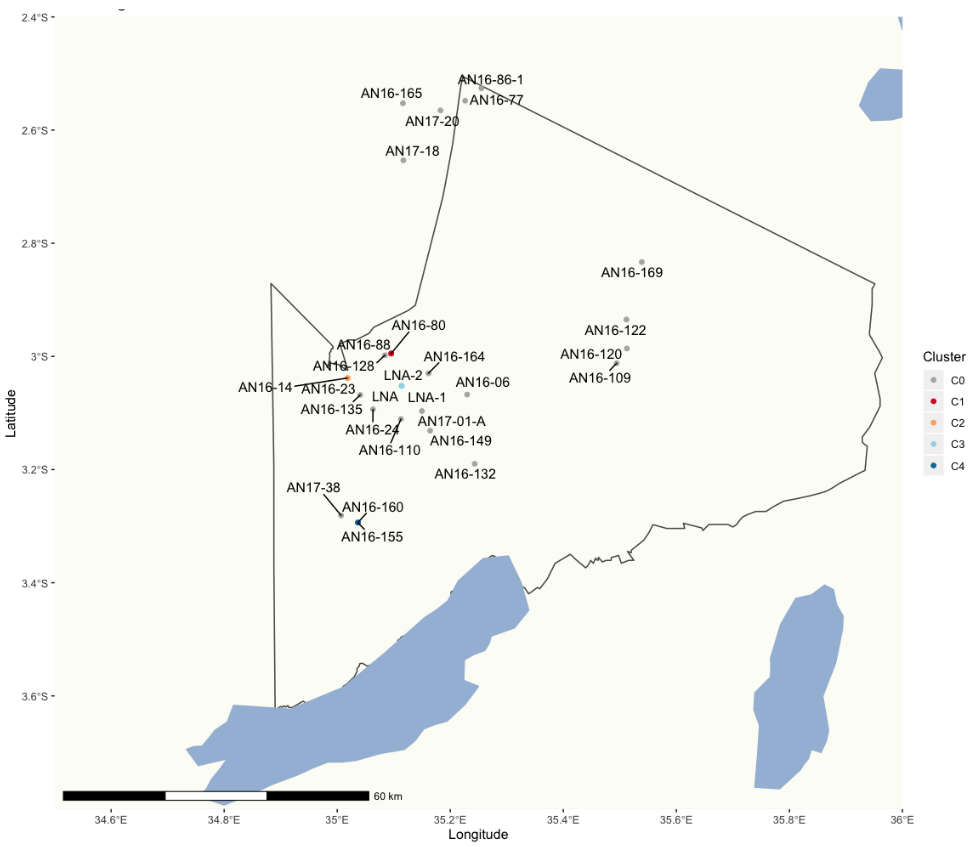

Fig S7. Locations of carcasses sampled within the Ngorongoro Conservation Area, northern Tanzania. Each carcass is assigned a unique identifier, included with the isolate metadata. Epidemiological clusters are the same as those explained for Fig. 3.

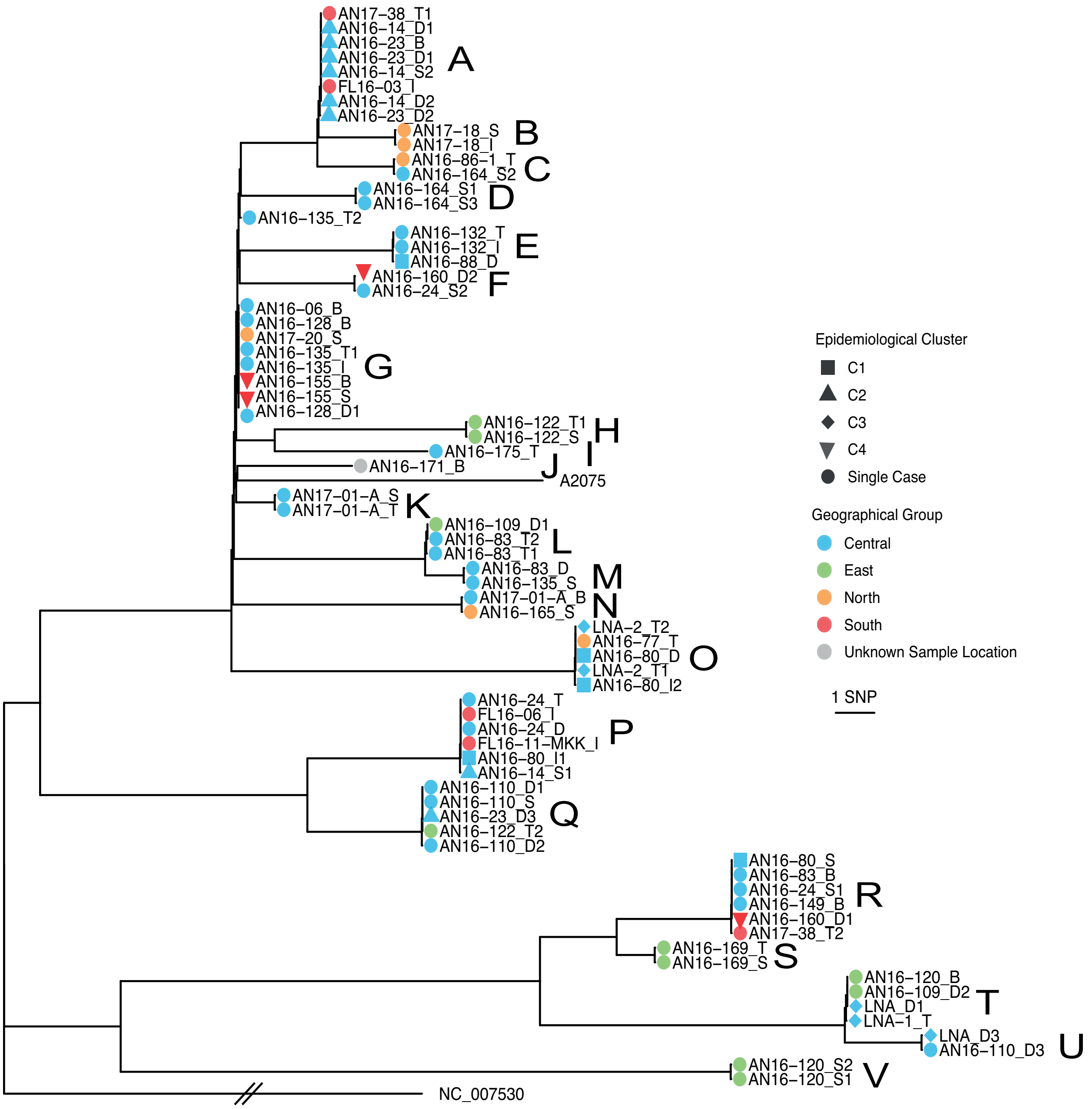

Fig S8. Phylogenetic relationship among *Bacillus anthracis* isolates from the Ngorongoro Conservation Area. Estimated through maximum likelihood, based on high quality core SNPs. Geographical groups and epidemiological clusters are the same as those explained for Fig 3. Each isolate is attributed to a carcass ID, with the sample type indicated following the underscore (B = blood, D = soil, I = insect, S = swab, T = tissue). A number follows the sample type if more than one isolate was sequenced from the same sample.

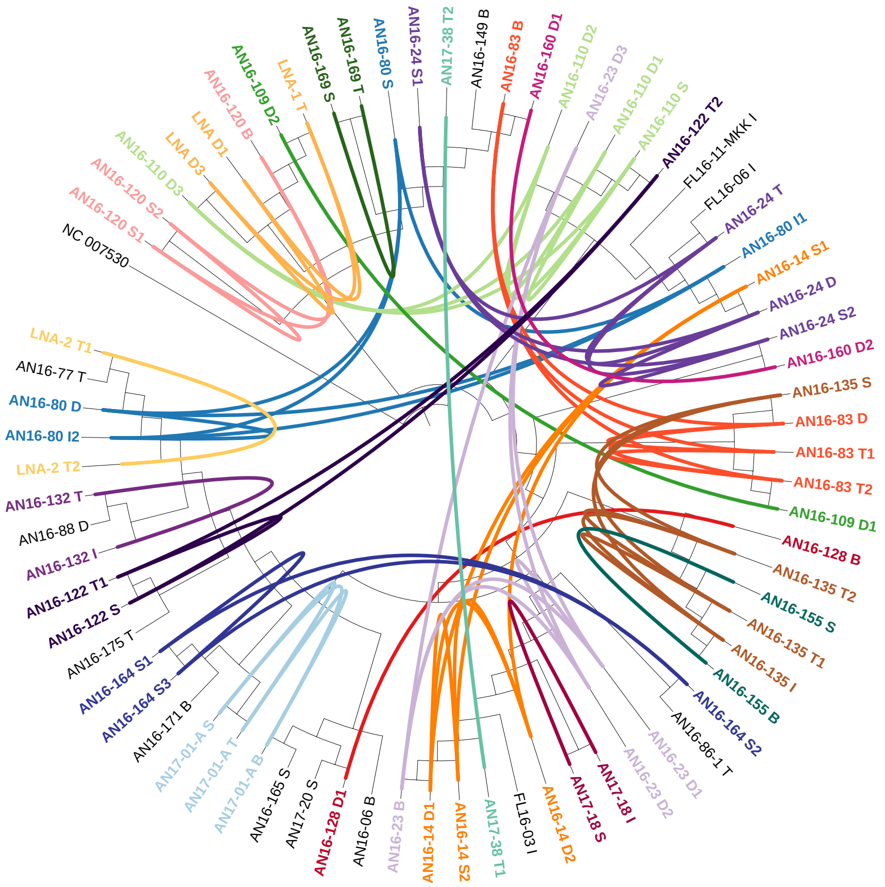

Fig S9. Within-host diversity of *Bacillus anthracis* isolated from livestock in the Ngorongoro Conservation Area of northern Tanzania. This circularized maximum likelihood tree – based on high quality core single nucleotide polymorphisms – is displayed as a cladogram (branch-lengths ignored). Isolates from the same carcass are shown in the same colour and linked by inner connecting lines. Isolates in black are singletons (i.e. only one isolate sequenced per carcass site). The figure was prepared using ITOL [2].

Supplementary Tables

Table S1. Publicly available *Bacillus anthracis* global genome sequences included to contextualize those from the Ngorongoro Conservation Area.

Strain	BioSample	BioProject	Assembly	Genetic Subgroup	Major canSNP groups from previous publications
2002013094	SAMN03174509	PRJNA238050	GCA_000832965.1	C.Br.001	C.Br.001
A1055	SAMN02435882	PRJNA10795	GCA_000167255.1	C.Br.001	C.Br.A1055
BF1	SAMN02469407	PRJNA171093	GCA_000295695.1	B.Br.003	B.Br.004
ANSES_00-82	SAMN02699416	PRJNA242332	GCA_000697555.2	B.Br.003	B.Br.004
CNEVA-9066	SAMN02435891	PRJNA10796	GCA_000167235.1	B.Br.003	B.Br.CNEVA
A0465	SAMN02470255	PRJNA27919	GCA_000181995.1	B.Br.003	B.Br.004
RA3	SAMN03075602	PRJNA238136	GCA_000832745.1	B.Br.003	B.Br.004
HYU01	SAMN02874036	PRJNA231762	GCA_000725325.1	B.Br.003	B.Br.001/002
SVA11	SAMN03081486	PRJNA217316	GCA_000583105.1	B.Br.003	B.Br.001/002
BA1035	SAMN03010427	PRJNA238135	GCA_000832725.1	B.Br.003	B.Br.001/002
Zimbabwe 89	SAMN02736952	PRJNA243517	GCA_000743805.1	B.Br.003	B.Br.001/002
Kruger B	SAMN02435884	PRJNA324	GCA_000167295.1	B.Br.003	B.Br.KrugerB
CZC5	SAMD00002586	PRJDB1571	GCA_000534935.1	A.Br.034	A.Br.005/006
K3	SAMN03010428	PRJNA238080	GCA_000832465.1	A.Br.034	A.Br.005/006
H9401	SAMN02603474	PRJNA49361	GCA_000258885.1	A.Br.047	A.Br.005/008 & 005/007
2000031038	SAMN03165124	PRJNA264742	GCA_000793565.1	A.Br.047	A.Br.005/008 & 005/007
K1129	SAMN03757460	PRJNA257008	GCA_001273105.1	A.Br.047	A.Br.007
SK-102	SAMN03012770	PRJNA238068	GCA_000832565.1	A.Br.047	A.Br.007
2000031709	SAMN03165119	PRJNA264742	GCA_000783035.1	A.Br.047	A.Br.007
2000031008	SAMN03165111	PRJNA264742	GCA_000793525.1	A.Br.047	A.Br.007
2000032892	SAMN03165115	PRJNA264742	GCA_000782905.1	A.Br.047	A.Br.007
CDC 684	SAMN02603931	PRJNA31329	GCA_000021445.1	A.Br.047	A.Br.007
2000031031	SAMN03165114	PRJNA264742	GCA_000782955.1	A.Br.047	A.Br.007
2000032951	SAMN03165125	PRJNA264742	GCA_000783115.1	A.Br.047	A.Br.007
Vollum 1B	SAMN03010433	PRJNA238082	GCA_000832445.1	A.Br.047	A.Br.Vollum
2000032967	SAMN03165126	PRJNA264742	GCA_000783135.1	A.Br.047	A.Br.007
2000032968	SAMN03165127	PRJNA264742	GCA_000783155.1	A.Br.047	A.Br.007
BA1015	SAMN03010426	PRJNA238204	GCA_000832665.1	A.Br.054	A.Br.003/004
2000031039	SAMN03165116	PRJNA264742	GCA_000782975.1	A.Br.054	A.Br.003/004
V770-NP-1R	SAMN03092715	PRJNA235226	GCA_000832785.1	A.Br.054	A.Br.003/004
A1039	SAMN02999502	PRJNA257008	GCA_000986915.1	A.Br.054	A.Br.003/004
K8215	SAMN03757461	PRJNA257008	GCA_001273125.1	A.Br.054	A.Br.003/004

A1075	SAMN02999503	PRJNA257008	GCA_000986935.1	A.Br.054	A.Br.003/004
2000032879	SAMN03165123	PRJNA264742	GCA_000783095.1	A.Br.075	A.Br.001/002
BFV	SAMN02736972	PRJNA243518	GCA_000742875.1	A.Br.075	A.Br.001/002
2000032979	SAMN03165129	PRJNA264742	GCA_000783195.1	A.Br.075	A.Br.001/002
ANSES_08-8_20	SAMN02693047	PRJNA242332	GCA_000697515.2	A.Br.075	A.Br.001/002
Sterne	SAMN02598266	PRJNA10878	GCA_000008165.1	A.Br.075	A.Br.001/002
A16	SAMN02641483	PRJNA40303	GCA_000512835.2	A.Br.081	A.Br.001/002
A16R	SAMN02641484	PRJNA40353	GCA_000512775.2	A.Br.081	A.Br.001/002
Shikan-NIID	SAMD00026520	PRJDB3549	GCA_002356575.1	A.Br.081	A.Br.001
Ames Ancestor	SAMN02603433	PRJNA10784	GCA_000008445.1	A.Br.081	A.Br.Ames
52-40-NIAH	SAMD00026914	PRJDB3563	GCA_001015025.1	A.Br.081	A.Br.001/002
44-NIAH	SAMD00026915	PRJDB3562	GCA_001015005.1	A.Br.081	A.Br.001/002
Han	SAMN02898318	PRJNA252784	GCA_000747375.1	A.Br.081	A.Br.001/002
A0389	SAMN02470266	PRJNA27917	GCA_000219895.1	A.Br.081	A.Br.001/002
9080-G	SAMN02951869	PRJNA224558	GCA_000558985.1	A.Br.014	A.Br.002/003
52-G	SAMN02951870	PRJNA224563	GCA_000559005.1	A.Br.014	A.Br.002/003
8903-G	SAMN02951868	PRJNA224562	GCA_000558965.1	A.Br.014	A.Br.002/003
A.br.Aust94	SAMN03757456	PRJNA257008	GCA_001273025.1	A.Br.014	A.Br.002/003
Ohio ACB	SAMN03010429	PRJNA238205	GCA_000832505.1	A.Br.014	A.Br.002/003
2000031027	SAMN03165113	PRJNA264742	GCA_000782895.1	A.Br.014	A.Br.002/003
K1285	SAMN03757454	PRJNA257008	GCA_001272985.1	A.Br.014	A.Br.002/003
Tsiankovskii-I	SAMN02436236	PRJNA17709	GCA_000181675.2	A.Br.105	A.Br.008/011
Cvac02	SAMN02898388	PRJNA252785	GCA_000747335.1	A.Br.105	A.Br.008/011
K2129	SAMN03757455	PRJNA257008	GCA_001273005.1	A.Br.118	A.Br.008/011
3154	SAMN02470705	PRJNA178572	GCA_000319695.1	A.Br.127	A.Br.008/011
3166	SAMN02470706	PRJNA178573	GCA_000319715.1	A.Br.127	A.Br.008/011
Pasteur	SAMN03024436	PRJNA238046	GCA_000832585.1	A.Br.127	A.Br.008/011
Smith 1013	SAMN02732407	PRJNA243516	GCA_000742315.1	A.Br.127	A.Br.008/011
A0157	SAMN03267488	PRJNA270580	GCA_000808075.1	A.Br.127	A.Br.008/011
Heroin Ba4599	SAMN02470707	PRJNA72893	GCA_000278385.1	A.Br.161	A.Br.008/011
ANSES_99-100	SAMN02699415	PRJNA242332	GCA_000697535.2	A.Br.133	A.Br.011/009
Carbosap	SAMN02910129	PRJNA231912	GCA_000732465.1	A.Br.133	A.Br.011/009
95014	SAMN02951914	PRJNA231913	GCA_000585275.1	A.Br.133	A.Br.011/009
A1144	SAMN02999504	PRJNA257008	GCA_000875715.1	A.Br.133	A.Br.011/009
Pollino	SAMN03296000	PRJNA273788	GCA_000831505.1	A.Br.144	A.Br.011/009
K0021	SAMN03757457	PRJNA257008	GCA_001273045.1	A.Br.144	A.Br.011/009
Sen2Col2	SAMEA2272511	PRJEB1516	GCA_000359425.1	A.Br.148	A.Br.011/009
Gmb1	SAMEA2272630	PRJEB1518	GCA_000359465.1	A.Br.148	A.Br.011/009
Sen3	SAMEA2272292	PRJEB1517	GCA_000359445.1	A.Br.148	A.Br.011/009
Canadian_bison	SAMN03202901	PRJNA238044		A.Br.009	A.Br.009
A0174	SAMN02470265	PRJNA27921	GCA_000182055.1	A.Br.009	A.Br.009

2000031006	SAMN03165110	PRJNA264742	GCA_000782875.1	A.Br.009	A.Br.009
A0193	SAMN02470282	PRJNA27923	GCA_000181915.1	A.Br.009	A.Br.009
Western N. Am. USA6153	SAMN02435885	PRJNA337	GCA_000167315.1	A.Br.009	A.Br.009
2000032832	SAMN03165122	PRJNA264742	GCA_000793545.1	A.Br.009	A.Br.009
2000032819	SAMN03165121	PRJNA264742	GCA_000783075.1	A.Br.009	A.Br.009
2000031765	SAMN03165120	PRJNA264742	GCA_000783055.1	A.Br.009	A.Br.009
2000032989	SAMN03165130	PRJNA264742	GCA_000783215.1	A.Br.009	A.Br.009

Table S2. Publicly available *Bacillus anthracis* sequence data (SRA) for Ancient A lineage to contextualize those from the Ngorongoro Conservation Area.

ID	Country	Source	Year	Genetic subgroup	Major canSNP group	Cluster (genotype based on Bruce et al., 2019)	NCBI accession numbers
A0026	England	equine	1992	A.Br.034	A.Br.005/006	3.1	SRR2968152 PRJNA302749 SAMN04283797
A2079	Tanzania	impala	1999	A.Br.034	A.Br.005/006	3.1	SRR2968188 PRJNA302749 SAMN04283798
A2075	Tanzania	baboon	1999	A.Br.034	A.Br.005/006	3.2	SRR2968187 PRJNA302749 SAMN04283799
A0017	Zambia	missing data	missing data	A.Br.034	A.Br.005/006	3.2	SRR2968151 PRJNA302749 SAMN04283800
CZC5	Zambia	hippopotamus	2011	A.Br.034	A.Br.005/006	3.2	BAVT01000000.1
DRR125654	Zambia	environment	2012	A.Br.034	A.Br.005/006	3.2	SAMD00113118
A0530	Botswana	elephant	missing data	A.Br.034	A.Br.005/006	3.3	SRR2968170 PRJNA302749 SAMN04283802
A0006	Australia	missing data	missing data	A.Br.034	A.Br.005/006	3.3	SRR2968150 PRJNA302749 SAMN04283803
DRR014741	Zambia	unknown	2011	A.Br.034	A.Br.005/006	3.3	DRR014741

Table S3. Identical isolates found from different geographical groups.

Carcass ID	Geographical group	Epidemiological cluster (if applicable)
AN17-01A	Central	
AN16-165	North	
AN16-80	Central	C1
LNA2	Central	C3
AN16-77	North	
AN16-110	Central	
AN16-23	Central	
AN16-122	East	
AN16-83	Central	
AN16-109	East	
AN16-80	Central	C1
AN17-38	South	
AN16-160	South	C4
AN16-24	Central	
FL-06	South	
FL-MKK	South	
AN16-14	Central	C2
AN16-23	Central	C2
FL16-03	South	
AN16-155	Central	
AN16-135	South	

Table S4. Identical or nearly identical isolates from carcasses sampled several months apart.

Groups of related isolates	Isolation dates	SNP differences	Time between sampling
AN16-86-1_T<br>AN16-164_S2	29-9-2016<br>25-1-2017	 0	4 months
AN16-165_S<br>AN17-01-A_B	26-1-2017<br>30-4-2017	 0	3 months
AN16-88_D<br>AN16-132_I<br>AN16-132_T	9-5-2016<br>21-3-2017	 <br>0	10 months
AN16-80_I2/D<br>LNA-2_T1/T2<br>AN16-77_T	10-5-2016<br>7-10-2016<br>9-10-2016	 <br>0	5 months
AN16-109_D1<br>AN16-83_T1/T2/D<br>AN16-135_S	18-7-2016<br>20-8-2016<br>20-10-2016	 0/0/1<br>1	3 months
AN16-80_I1<br>AN16-14S<br>AN16-24_D/T	10-5-2016<br>16-9-2016<br>26-9-2016	 <br>0	4 months
AN16-110_D3<br>LNA_D3	30-6-2016<br>7-10-2016	 0	3 months

SNP: single nucleotide polymorphism

Table S5. Single nucleotide polymorphism (SNP) distances among pairs of isolates sampled from simulated within-host populations of *Bacillus anthracis*. Proportion of pairs of isolates in evolved populations with different SNP distances across varying initial inoculum size (dose), sampled in generations 20 and 25, and mean SNP differences across sampled pairs.

	Generation 20					Generation 25[1]				
Dose	0 (%)	1 (%)	2 (%)	3 (%)	Mean SNP distance	0 (%)	1 (%)	2 (%)	3 (%)	Mean SNP distance
1	86.1	12.7	1.16	0.06	0.15	82.5	15.8	1.49	0.16	0.19
2	85.7	13.3	0.82	0.10	0.15	82.6	16.0	1.64	0.12	0.19
5	85.3	13.6	1.11	0.05	0.16	81.2	17.1	1.56	0.07	0.20
10	86.1	12.7	1.18	0.05	0.15	82.2	15.9	1.76	0.12	0.20
20	84.1	14.6	1.20	0.02	0.17	81.3	16.8	1.70	0.16	0.21
50	85.0	13.7	1.26	0.02	0.16	82.9	15.7	1.29	0.14	0.19
100	84.7	13.9	1.32	0.04	0.17					

[1] Values for generation 25 with dose 50 are based on a small number of simulations (7) only.

File S1. Supplementary methods and results.

*Note: All isolate metadata, including carcass ID, species of animal sampled, location of sampling, and sequence quality metrics are available at:

http://dx.doi.org/10.5525/gla.researchdata.1217

Materials & Methods

Study area

The population of the Ngorongoro Conservation Area (NCA) is comprised mostly of Maasai pastoralists. The size was based on a population estimate of just over 70,000 inhabitants in 2012 and an annual growth rate of 2.7%. Given this area's conservation status, in addition to livestock, people live in close proximity with various wildlife species.

Research and ethical approval

Material and data transfer agreements were established as part of the research approvals. This study complied with Tanzania's national access measures for genetic material. Approval and permission to access communities were also obtained from relevant local authorities. Verbal and/or written informed consent was obtained from all owners of livestock sampled, with verbal consent obtained in lieu of written consent where participants preferred. Both verbal and written consent had been approved by the ethical committees.

Sample collection and handling during shipping

Anthrax was suspected in livestock for any acute mortality where the animal had appeared healthy until the time of death. Terminal bleeding from natural orifices was variably observed. Samples were triple-bagged, with the inner container first disinfected with 10,000 ppm sodium hypochlorite reconstituted from Haz-Tab tablets (Guest Medical, UK). GPS coordinates were taken for samples collected later in the study; for earlier samples, coordinates were estimated by

having the field team identify the sampling location on a map of the study area and subsequently plotting these points using Google Earth. When available, GPS coordinates are provided in with the isolate metadata to three decimal places to ensure participant anonymity.

Aliquots from a total of 278 samples from 109 carcasses were shipped to the University of Pretoria, South Africa, for selective culture and DNA extraction from *B. anthracis* isolates. Of the samples sent, about half had previously tested positive by qPCR (142 samples from 63 carcasses). Upon arrival in South Africa, sample tubes were sprayed with disinfectant then heat inactivated for 72 °C for 20 minutes at the Transboundary Animal Diseases section of the Onderstepoort Research Institute as per South African governmental regulations in the control of Foot and Mouth Disease.

Bacterial isolation

Tissues

The transit time and heat treatment had resulted in advanced putrefaction and some fungal contamination in the specimen tubes. A salt solution consisting of 900 µL NaCl (5mg/mL) was added to the tubes and left overnight to inhibit the vegetative fungi. The specimens were washed with distilled water and the supernatant discarded four times with successive centrifuging at 8000 XG for 20 min in between. The clean tissue pellet was homogenized in 500 µL phosphate buffered saline (PBS) with glass beads using a single low impact cycle in a Precellys® Tissue homogenizer (Bertin GMBH, Frankfurt Germany).

Swabs

The dry swabs were moistened with 350 µL Dulbecco's saline (fortified with additional 1 M calcium carbonate) for 24 hours. The swabs were softened by the saline solution and the solute took on the appearance of diluted blood after vortexing.

Blood

Blood samples were diluted in 250 µL PBS and were heat treated at 65 °C for 10 minutes to inhibit competition from heat-sensitive bacteria.

Insects

The insects were identified under a stereoscope while being placed in sterile 1.5 mL Eppendorf tubes filled with 350 µL PBS. A homogenizing pestle (Lasec, South Africa) was used to homogenize the sample for plating.

Sub-Culture and DNA extraction

Only grey-white, ground-glass textured, non-hemolytic colonies were selected for subculture from sheep blood agar (SBA) (within 24 hours) and dome shaped, rough white colonies were selected from PET (within 48 hours). Differentiating morphology characteristics among suspect isolates included (i) pronounced "medusa head" edges around the colony forming unit; (ii) an uncharacteristic tacky texture when touched with the bacteriologic loop; and (iii) a larger colony diameter than other colony forming units on the same plate.

Once pure, single colonies could be isolated after passage, species was confirmed using a 10 µg penicillin disc (Oxoid) and 10 µL of diagnostic Gamma phage ($5x10^9$ pfu/mL); colonies were identified as *B. anthracis* when demonstrating both penicillin and γ-phage sensitivity after overnight incubation at 37°C, as well as testing positive by qPCR for the protective antigen *pag* gene (BAPA) [3]. DNA extraction for qPCR confirmation was performed by harvesting all material on the purity plate in 5 mL of PBS and crude boiled at a 110 °C for 15 minutes. The boiled solution was then centrifuged at 6000 G for 30 min, reserving the supernatant.

To extract genomic DNA for sequencing, a loopful of bacterial colonies from the purity plates was used as the input for the Bioline Isolate II Genomic DNA kit (Meridian Biosciences, UK)

with 2 hour 37°C incubation in 20 mg/mL lysozyme (Fluka) solution according to the manufacturer's instructions for Gram-positive bacteria.

qPCR

The qPCR for BAPA [3–5] was performed with 2.5 µL DNA in 1x FastStart™ Taq DNA Polymerase mastermix (Roche®) and 0.5 µM of each primer along with 0.2 µM of each FRET probe (Tib MolBiol GmbH) in a final volume of 20 µL. The PCR conditions on a LightCycler™ Nano (Roche®) consisted of an initial cycle at 95 °C for 10 minutes, slope at 20 °C/second, followed by 40 cycles of 95 °C for 10 seconds; 57 °C for 20 second; 72 °C for 30 second, slope 20 °C/second with one single signal acquisition at the end of annealing cycle. Denaturation at 95 °C for 3 seconds with a slope 20 °C/second; 40 °C for 30 seconds, slope 20 °C/second; 80 °C for 3 seconds at a slope of 0.1 °C/second with continuous acquisition of the signal. Cooling to 40 °C for 30 second, slope 20 °C/second [3, 5]. Plates and CFU that were negative for both BAPA as well as phage sensitivity were excluded for final nucleic acid extraction selection.

Additional quality control

DNA was extracted from 96 isolates and shipped to the UK for further quality control and subsequent sequencing. DNA extracts from 75 isolates from 33 carcasses were submitted for sequencing; these were selected based on having sufficiently high concentration as measured by Qubit (≥1 ng/µl) and lower Ct values (< 25) on qPCR (i.e. higher concentrations of *B. anthracis* DNA); Ct values of DNA extracts from sequenced isolates ranged from 9-21.

Bioinformatics

SNP positions identified using VarScan from all individual NCA samples, in addition to A2075, were merged into a single dataset using an in-house python script, resulting in a total of 721 unique variant sites across isolates. Subsequently, read mapping metrics files for the detected set

of variant sites were generated for individual isolates using bam-readcount tool v.0.8.0 (https://github.com/genome/bam-readcount). Variant sites were removed based on the following rules: 1) occurred in phage regions as detected by PHASTER [6]; 2) occurred in repetitive/homologous genomic regions; 3) more than 3 isolates at a particular site had prevalent base frequency below 89% or/and read depth below 4. In the final alignment file, N character was assigned to sites with prevalent base frequency below 89%, and gap character (-) was assigned to sites having read depth below 4. The resulting filtered alignment file consisted of 611 positions, of which 437 were monomorphic among isolates from the NCA and A2075 (i.e. all isolates had the same allele that differed from the reference). For the purposes of phylogenetic analysis, all gaps and N characters were removed from the alignment file, leaving 499 sites, of which 374 were monomorphic and 125 polymorphic.

Simulation modelling

One hundred simulations running for 25 generations were performed for inoculum sizes one, two, five, and ten. Due to computational restrictions, only 50 simulations running for 25 generations were performed for dose 20. One hundred simulations running for 20 generations were performed for doses 50 and 100, while an additional 7 simulations with an initial dose of 50 were run for 25 generations.

Results

Bacterial Isolation

Of the 278 specimens processed for culture, 122 yielded suspect *B. anthracis* colonies, while the remainder had almost no growth on either the *B. anthracis* selective and non-selective media. After testing the suspect colonies with penicillin, Gamma phage and qPCR for the protective

antigen *pag* gene, 73 samples (96 individual colonies) had results confirmatory of *B. anthracis* isolates. This included isolates from two carcasses from which none of the samples (soil, tissue and blood) had previously tested positive on qPCR using established Ct value cut-offs [7]. After long-term sample storage at ambient temperature, *B. anthracis* isolation was least successful from whole blood, with hardly any bacterial growth after the heat treatment. The tissue, insect, swabs and soil all performed similarly in producing viable isolates, although the swabs had almost pure *B. anthracis* plates for some of the samples, making it the most suitable sample type for bacterial isolation under this type of collection and storage conditions. The 122 specimens that yielded any growth at all were almost exclusively made up of a combination of *B. endophyticus, B. cereus, B. pumilus, B. megaterium, B. subtilis, B. thuringiensis* and *B. anthracis* representing the hardiest spore formers within the specimens.

Several *B. anthracis* isolates were obtained from flies captured on or around carcasses, most of which were *Muscidae* species. While speciation was difficult in some cases due to damaged wings and/or proboscis, species were predominantly *Musca crassirostris* and to a lesser extent *Musca domestica.* No efforts were made to determine whether the spores were on the surface or in the gut of the flies. In certain ecosystems, flies are thought to play an important role as mechanical vectors, spreading spores onto leaves which may become a source of infection for browsing animals [8, 9]. The role of biting flies as a source of *B. anthracis* infection is less well defined [3].

Sequence quality

GC content of the raw reads was unexpectedly low (<34; n=1) or high (>36; n=5), and/or total *de novo* assembly length was above the expected length of ~5.5 MB (n=13), and/or there were a high number of contigs (>1000; n=11), indicative of challenges with assembly that could be

related to mixed culture. A total of 16 isolates had one or more of these issues (see isolate metadata). Strict filtering criteria were implemented during reference-based mapping to address this issue.

Simulation modelling

After 20 generations and across 100 simulations, a population with a starting dose of 1 (n = 524,288) had an average of 2,258 (range 2,140 to 2,364) unique SNP profiles, i.e. that differed by at least one SNP. This rose to 71,845 (range 71,049 to 72,610) after 25 generations. When averaging across simulations, inoculum size (infectious dose) did not significantly influence the proportion of the population identical to the infecting genome, or that differed by 1 or 2 SNPs. Across inoculum sizes, the proportion of genomes identical to the infecting dose in our simulations tended to remain high, and while genomes differing by a maximum of 5 (doses 1 and 2) and 6 (doses 5 – 100) SNPs were observed, these were incredibly rare and therefore unlikely to be sampled; the majority of variant genomes differed from the inoculum genome by a single SNP. Greater variability around the average proportion of the population with different numbers of SNPs was observed in populations simulated from the smaller inoculum sizes due to increased impact of stochasticity in early generations. For example, in a single simulation with an inoculum size of 1, a mutation emerged in the first replication and therefore no genomes identical to the infecting dose were observed in further generations (Fig. S7-A), while in some simulations initiated with an infectious dose of 2, two major variants separated by a single SNP were both present at fairly even proportions (Fig. S8-B).

Proportions of sampled pairs of genomes from the simulated populations with different numbers of SNP differences are summarized in Table S5, which also shows the mean SNP distance averaged across sampled pairs. This mean SNP distance ranged from 0.15 (dose = 1) to 0.17

(dose = 100). While pairwise distances ranging from 0 to 4 were observed, this only included 33 observations of 3 SNP distance (0.05%) and 5 observations of 4 SNP distance (0.008%).

The relationship between higher dose and higher mean SNP distance was statistically significant ($p < 0.005$) though the estimated effect size was small (+0.0014 per extra 10 genomes in dose) and resulted in only a slight increase in the chance of drawing pairs of genotypes with greater SNP distances. For example, the proportion of samples with SNP distances of 2 or more increased only from 1.22% with an inoculum size of 1, to 1.37% with an inoculum of 100 genomes.

At least 50 simulations initiated with inoculums of 1, 2, 5 10 and 20 bacteria were run for an additional 5 generations. The mean SNP distance between pairs sampled in generations 20 to 25 tended to increase slightly (Fig. 6D), by around 0.0085 per generation ($p < 0.0001$). This gradual increase resulted in the proportion of samples with a SNP distance of 2 rising from 1.15% in generation 20 to 1.74% in generation 25. In 270,000 pairs sampled in generations 21 to 25, a SNP distance of 4 was observed only 8 times and a SNP distance of 5 was sampled once (in a population simulated from an infectious dose of 10 sampled in the 24th generation). Such an observation is incredibly rare as it requires, in the most likely scenario, sampling 2 genotypes differing from the inoculum by 2 and 3 non-shared mutations.

References

1. **Hsieh TC, Ma KH, Chao A**. iNEXT: an R package for rarefaction and extrapolation of species diversity (Hill numbers). *Methods Ecol Evol* 2016;7:1451–1456.

2. **Ciccarelli FD, Doerks T, Mering C von, Creevey CJ, Snel B, *et al.*** Toward automatic reconstruction of a highly resolved Tree of Life. *Science* 2006;311:1283–1287.

3. **WHO**. *Anthrax in Humans and Animals*. 4th Ed; 2008.

4. **Bell CA, Uhl JR, Hadfield TL, David JC, Meyer RF, *et al.*** Detection of Bacillus anthracis DNA by LightCycler PCR. *J Clin Microbiol* 2002;40:2897–2902.

5. **Ellerbrok H, Nattermann H, Ozel M, Beutin L, Appel B, *et al.*** Rapid and sensitive identification of pathogenic and apathogenic Bacillus anthracis by real-time PCR. *FEMS Microbiol Lett* 2002;214:51–59.

6. **Arndt D, Grant JR, Marcu A, Sajed T, Pon A, *et al.*** PHASTER: a better, faster version of the PHAST phage search tool. *Nucleic Acids Res* 2016;44:W16–W21.

7. **Aminu OR, Lembo T, Zadoks RN, Biek R, Lewis S, *et al.*** Practical and effective diagnosis of animal anthrax in endemic low-resource settings. *PLOS Neglected Tropical Diseases* 2020;14:e0008655.

8. **Fasanella A, Scasciamacchia S, Garofolo G, Giangaspero A, Tarsitano E, *et al.*** Evaluation of the house fly Musca domestica as a mechanical vector for an anthrax. *PLoS ONE* 2010;5:e12219.

9. **Blackburn JK, Van Ert M, Mullins JC, Hadfield TL, Hugh-Jones ME**. The necrophagous fly anthrax transmission pathway: empirical and genetic evidence from wildlife epizootics. *Vector Borne Zoonotic Dis* 2014;14:576–583.
